# A semi-automatic technique to quantify complex tuberculous lung lesions on ^18^F-fluorodeoxyglucose positron emission tomography/computerised tomography images

**DOI:** 10.1186/s13550-018-0411-7

**Published:** 2018-06-25

**Authors:** Stephanus T. Malherbe, Patrick Dupont, Ilse Kant, Petri Ahlers, Magdalena Kriel, André G. Loxton, Ray Y. Chen, Laura E. Via, Friedrich Thienemann, Robert J. Wilkinson, Clifton E. Barry, Stephanie Griffith-Richards, Annare Ellman, Katharina Ronacher, Jill Winter, Gerhard Walzl, James M. Warwick, the Catalysis Biomarker Consortium, Stephanus T. Malherbe, Patrick Dupont, Ilse Kant, Katharina Ronacher, Magdalena Kriel, André G. Loxton, Ray Y. Chen, Laura E. Via, Friedrich Thienemann, Robert J. Wilkinson, Clifton E. Barry, Stephanie Griffith-Richards, Annare Ellman, Jill Winter, Gerhard Walzl, Nelita Du Plessis, Caroline G. G. Beltran, Lani Thiart, Gerard Tromp, Lance A. Lucas, Bronwyn Smith, Kim Stanley, David Alland, Shubhada Shenai, Lori E. Dodd, James M. Warwick

**Affiliations:** 1 DDST-NRF Centre of Excellence for Biomedical Tuberculosis Research and South African Medical Research Council Centre for Tuberculosis Research, Cape Town, South Africa; 2 Division of Molecular Biology and Human Genetics, Faculty of Medicine and Health Sciences, Stellenbosch University, Cape Town, South Africa; 3 Department of Neurosciences, Laboratory for Cognitive Neurology, KU Leuven, Belgium; 4 Division of Nuclear Medicine, Department of Medical Imaging and Clinical Oncology, Faculty of Medicine and Health Sciences, Stellenbosch University, Cape Town, South Africa; 5 Tuberculosis Research Section, Laboratory of Clinical Infectious Diseases, Division of Intramural Research, National Institute of Allergy and Infectious Diseases, National Institutes of Health, Bethesda, MD USA; 6 Wellcome Centre for Infectious Disease Research in Africa, Institute of Infectious Disease and Molecular Medicine, Faculty of Health Science, University of Cape Town, Observatory, 7925 Republic of South Africa; 7 Department of Medicine, Faculty of Health Science, Groote Schuur Hospital, University of Cape Town, Cape Town, South Africa; 8 The Francis Crick Institute, Midland Road, London, NW1 2AT UK; 9 Department of Medicine, Imperial College London, London, W2 1PG UK; 10 Division of Radiodiagnosis, Department of Medical Imaging and Clinical Oncology, Faculty of Medicine and Health Sciences, Stellenbosch University, Cape Town, South Africa; 11 Translational Research Institute, Mater Research Institute – The University of Queensland, Brisbane, QLD Australia; 12 Catalysis Foundation for Health, Emeryville, CA USA

**Keywords:** ^18^F-fluorodeoxyglucose (FDG) positron emission tomography (PET)/computed tomography, Tuberculosis, Image analysis, Lesion segmentation, Lesion quantification

## Abstract

**Background:**

There is a growing interest in the use of ^18^F-FDG PET-CT to monitor tuberculosis (TB) treatment response. However, TB causes complex and widespread pathology, which is challenging to segment and quantify in a reproducible manner.

To address this, we developed a technique to standardise uptake (Z-score), segment and quantify tuberculous lung lesions on PET and CT concurrently, in order to track changes over time. We used open source tools and created a MATLAB script. The technique was optimised on a training set of five pulmonary tuberculosis (PTB) cases after standard TB therapy and 15 control patients with lesion-free lungs.

**Results:**

We compared the proposed method to a fixed threshold (SUV > 1) and manual segmentation by two readers and piloted the technique successfully on scans of five control patients and five PTB cases (four cured and one failed treatment case), at diagnosis and after 1 and 6 months of treatment. There was a better correlation between the Z-score-based segmentation and manual segmentation than SUV > 1 and manual segmentation in terms of overall spatial overlap (measured in Dice similarity coefficient) and specificity (1 minus false positive volume fraction). However, SUV > 1 segmentation appeared more sensitive. Both the Z-score and SUV > 1 showed very low variability when measuring change over time. In addition, total glycolytic activity, calculated using segmentation by Z-score and lesion-to-background ratio, correlated well with traditional total glycolytic activity calculations. The technique quantified various PET and CT parameters, including the total glycolytic activity index, metabolic lesion volume, lesion volumes at different CT densities and combined PET and CT parameters. The quantified metrics showed a marked decrease in the cured cases, with changes already apparent at month one, but remained largely unchanged in the failed treatment case.

**Conclusions:**

Our technique is promising to segment and quantify the lung scans of pulmonary tuberculosis patients in a semi-automatic manner, appropriate for measuring treatment response. Further validation is required in larger cohorts.

## Background

Positron emission tomography/computerised tomography (PET-CT) is well established in the diagnostic workup, treatment planning and response assessment of cancer and various inflammatory and infectious diseases [1, 2]. The most commonly used PET tracer is ^18^F-fluorodeoxyglucose (FDG). It reflects glucose metabolism and shows increased uptake in areas of inflammation. PET scanners measure the radiopharmaceutical concentration in tissue [3–5].

Uptake intensity in tissue is variable and influenced by numerous patient-, timing- and equipment factors, which is why the lesion-to-background ratio is often considered a more robust measure than absolute uptake [6]. Several semi-quantitative measurements of PET voxel intensity have been developed, of which standardised uptake value (SUV), is most commonly used. It compensates for variation in body size, injected activity and radioactive decay.

The metabolic lesion volume (MLV), maximum and mean SUV within the lesion (SUV_max_ and SUV_mean_) and the total glycolytic activity (TGA = MLV × SUV_mean_) are the PET parameters most commonly used for lesion quantification [3, 4, 7–9].

The borders of the MLV can be delineated visually or using various semi-automated techniques, however no single technique has proven optimal for all applications [6]. In most cases lesion delineation is still performed manually, based on visual interpretation of PET or CT images. This is prone to inter- and intra-operator variation, especially for PET due to its lower spatial resolution and inherent noise [8–15]. Multiple methods are used and proposed to decrease variation in lesion segmentation. These include using reference values to normalise the lesion- to- background uptake intensity by comparison to liver or mediastinal blood pool uptake [16–18] and the use of automated segmentation techniques. Automated segmentation techniques include thresholding techniques, gradient-based techniques and stochastic- and learning-based computerised methods [6]. Thresholds may be fixed or adaptive. Adaptive thresholds utilises image parameters, such as lesion-to-background ratio, mean background intensity and estimated lesion intensity in algorithms to define the threshold.

Some studies also focused on the quantification of images from CT scanners, which measure the density of anatomical structures and lesions in Hounsfield units (HU). It is not a functional scan and is less prone to interscan variability. Only a few studies have evaluated densometric quantification of CT scans in diffuse lung disease; however, it appears to be reproducible and correlate well with other disease markers [19–22]. Joint segmentation of fused PET and CT images has shown improved robustness compared to methods using only data from PET [23].

The shortcomings of sputum culture to accurately indicate when TB treatment has achieved sterilising cure [24–26] escalate the cost to develop improved TB treatment options. This has led to a growing interest to use PET-CT imaging to monitor TB treatment response [27–29]. In animal models, FDG PET-CT has been used to accurately describe disease progression and response to treatment in pulmonary tuberculosis (PTB) [30–35]. Human studies have also shown PET-CT of promise to monitor the effect of treatment using simple descriptive techniques [27, 31, 36–41]. We recently reported PET-CT findings in PTB patients, before, during and after therapy, and found strikingly complex and heterogeneous lesion responses [42].

**Fig. 1:**
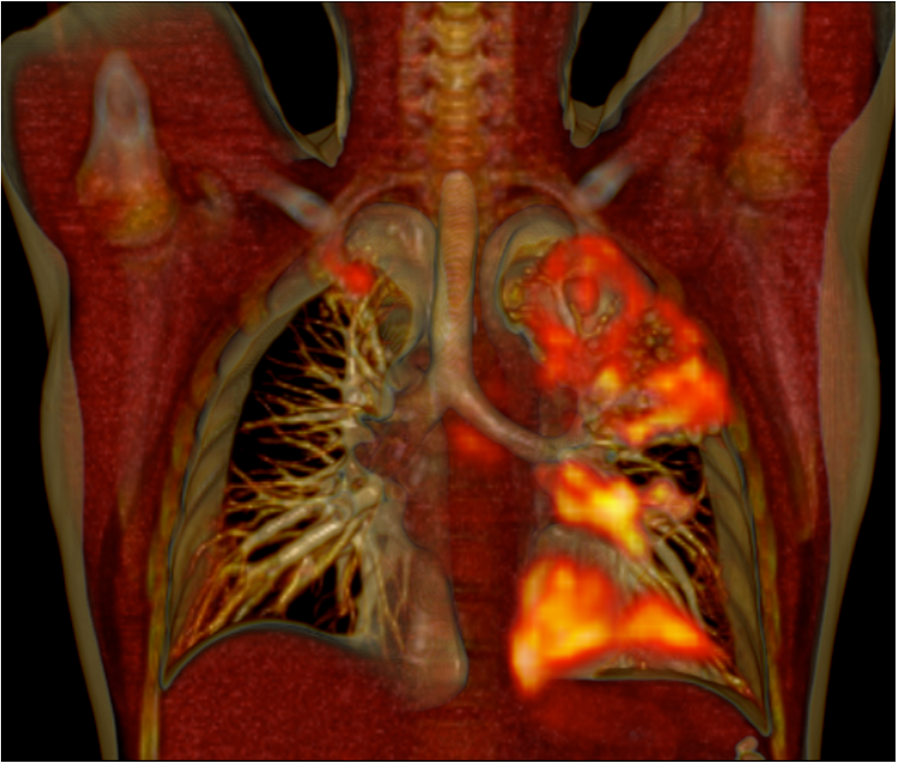
3D rendered anterior view of fused ^18^F-FDG-PET-CT scan, performed at diagnosis on a patient with sputum culture positive pulmonary tuberculosis. It shows a wide distribution of lesions with complex morphology, including a large cavity in the left upper lobe with surrounding nodular infiltrates and patches of consolidation in the left lower lobe

Reproducible segmentation and quantification becomes particularly important in diseases with heterogeneous morphology, vague borders and multi-focal distribution throughout an organ or system, such as TB or sarcoidosis [31, 41] (example shown in Fig. 1). This is especially important when accurate tracking of changes over time is required. In most animal model PET-CT studies, the authors used manual lesion delineation to track changes throughout the lungs, or in individual lesions [30–35]. Two separate human studies implemented whole lung quantification of PET (using fixed thresholds of SUV > 1 and SUV > 2, respectively) and semi-quantitative CT reader scores [31, 41]. These found that quantified PET images were more robust than reader-based CT scores, and seemed to accurately measure changes in disease burden over time. To account for the spatially complex lesions, two studies successfully utilised computer-aided segmentation, respectively, based on affinity propagation and both interactive region growing and adaptive thresholding, in PET images of TB-infected small animal models [43, 44].

In this report, we describe a semi-automated, voxel-based technique developed for both PET and CT quantification to standardise and segment widespread and heterogenous lesions throughout the lung and reproducibly measure disease burden over time.

## Methods

### Study design

The method was optimised on a training set of 15 lesion-free lung scans obtained from patients undergoing PET-CT scans for non-pulmonary clinical indications, as well as scans from five PTB patients showing residual lung lesions after treatment.

As a proof of concept, we applied the methodology to quantify disease burden on an independent test set of PET-CT scans from five controls and serial scans from the first five patients participating in the PTB cohort study, mentioned above [42]. The controls were from the same communities (Cape Town, South-Africa), and had contact with PTB patients, but were sputum culture negative for *Mycobacterium tuberculosis* and had no active lesions visible on PET-CT scan. PTB cases were all diagnosed with drug-sensitive TB strains and were HIV negative. Cases underwent scans at time-points within 1 week from initiation of treatment (Dx), after 1 month of treatment (M1) and after 6 months of treatment (M6), corresponding with the duration of standard TB therapy. At the end of treatment, four of the PTB cases were sputum culture negative and classified as cured by healthcare providers in charge of treatment, while one was still sputum culture positive and diagnosed as a failed treatment outcome. The study design is summarised in Table 1.

**Table 1:** Study design for method development and pilot application

	Training set		Test set	
Group	Negative controls	Positive controls	Negative controls	PTB cases
	*n* = 15	*n* = 5	*n* = 5	*n* = 5
PET-CT indication	Clinical	Observational cohort	Observational cohort	Observational cohort
Clinical background	Diagnostic work-up	PTB patients, after treatment	PTB contacts	PTB patients on treatment,
Scan time-points	Single scan	Single scan, after TB treatment	Single scan	Baseline, month 1 and month 6 of treatment
Lung scan findings	Lesion free	Minimal to mild intensity lesions after TB treatment	Lesion free	Extensive lesions
Main function of inclusion	Optimising *Z*-score threshold specificity	Optimising *Z*-score threshold sensitivity	Testing specificity	Pilot application

#### PET-CT imaging

PET-CT scanning was performed with a Philips Gemini Big Bore time-of-flight scanner according to internationally accepted guidelines [45]. Patients fasted for 6 h before FDG administration, but were encouraged to hydrate well. According to body weight, participants received 185–259 MBq of ^18^F-FDG intravenously 60 min before scan acquisition. PET images were reconstructed to 4 × 4 × 4 mm voxels using an iterative algorithm including time-of-flight information and corrections for random events, scatter, deadtime, attenuation and decay. The CT scan parameters were set at 120 kV, 100 mAs, without dose modulation with 1.17 × 1.17 mm pixels and a 3 mm slice thickness.

#### Pre-processing

We exported images from the PET-CT workstation in DICOM format and converted to ANALYSE file format [46] using MRIConvert [47]. To ensure reproducibility and facilitate direct comparison, we co-registered each patient’s follow-up scans with the baseline CT, using Statistical Parametric Mapping (SPM8) [48] with MATLAB 2013b (Mathworks Inc.). During the co-registration (using within-subject rigid-body model), CT images were re-sliced to the voxel matrix of the corresponding PET scan (using trilinear interpolation). This allowed direct voxel-based comparison of each patient’s PET and CT images.

#### Lung masks for PET and CT

**Fig. 2:**
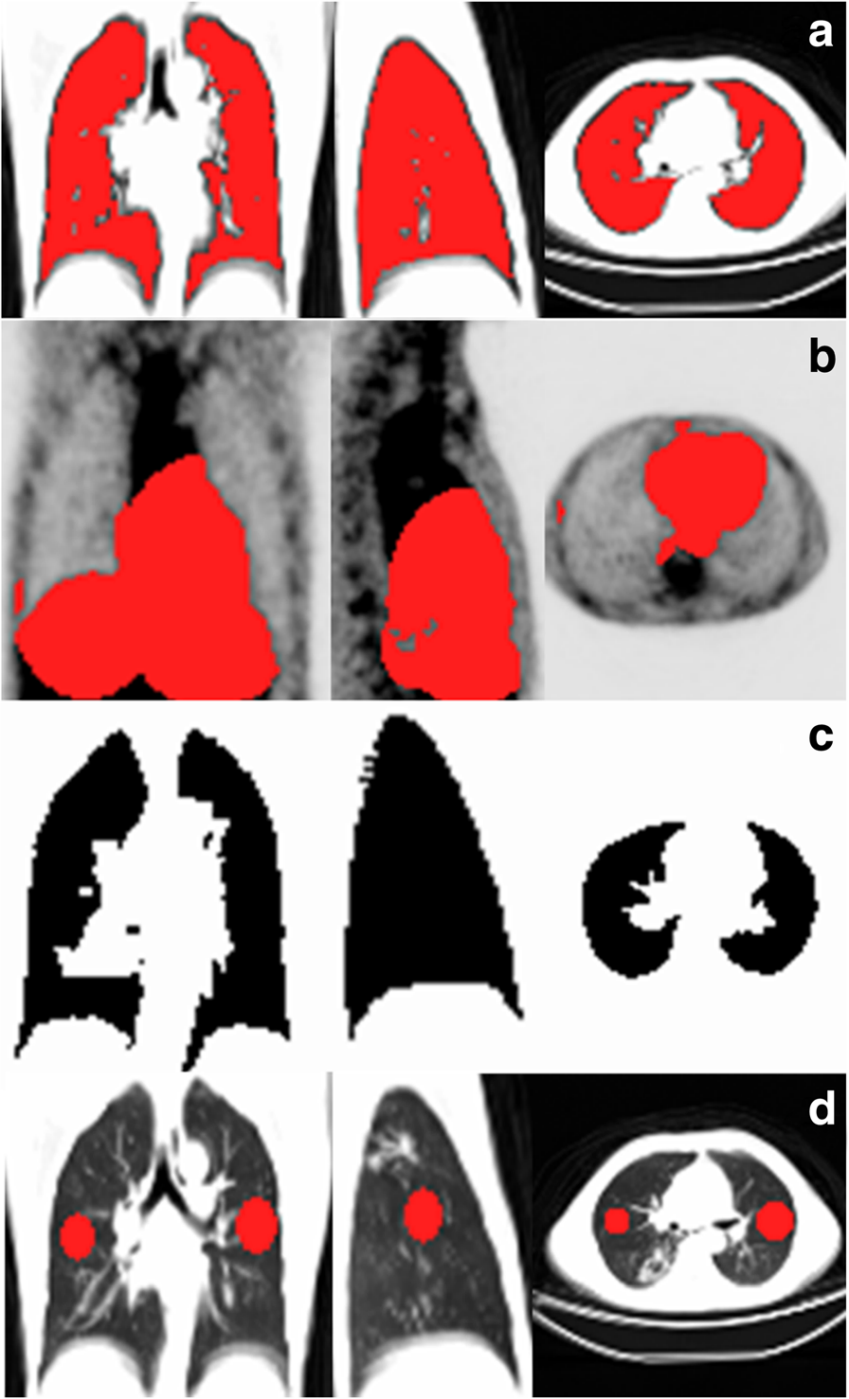
Example of volumes of interest (in red) required for quantification. **a** Lung mask, on the combined CT images from baseline and follow-up scans. **b** Volume of structures surrounding the lung affecting motion misregistration (usually the mediastinum, liver and spleen) drawn on the overlaid PET images. We then deleted overlapping areas on the lung mask to leave only areas unaffected by misregistration. **c** Binary image representing the final lung mask. **d** Reference volumes of normal lung tissue, represented by two spheres in opposite lungs or ipsilateral lobes. It is also drawn on the overlaid CT images, but viewed alongside the PET images to ensure that it represents lesion-free lung

For each series of co-registered CT studies, we generated a volume of interest (VOI) as a lung mask (Fig. 2a), in MRICro version 1.39 [49] on overlaid CT images, using the 3D region-growing, gradient-based tool. This allowed setting the origin, radius and difference from origin and edge gradient on the overlaid CT images. We combined this with manual corrections, to avoid the exclusion of dense lesions extending into the pleura from the VOI. The lung mask excluded the lung hila and main pulmonary vessels, but included smaller vessels. As a final adjustment, we created a VOI of organs around the lungs that included in the lung fields by misregistration [usually the liver, spleen and mediastinum (Fig. 2b)], with the region-growing tool on the PET scans, and then deleted overlapping voxels from the lung mask. We converted the mask to a binary image and filled any holes in the mask, using ImageJ software [50] (Fig. 2c).

#### PET quantification: background reference

We selected two areas of normal lung parenchyma (NL) as a reference VOI to standardise uptake in lung voxels (Fig. 2d) [50]. Each NL volume consisted of spheres 15 to 25 mm in diameter that visually appeared lesion-free on all co-registered scans. To enhance the representivity of the sample, the spheres were selected in opposite lungs or in some cases in different ipsilateral lobes, depending on the distribution of lung pathology.

#### PET image quantification

We then quantified the pre-processed series of PET-CT images, using a MATLAB script developed in-house. The script quantified disease burden from the PET images by assigning a *Z*-score to all voxel counts within the lung mask based on the equation below:

$$ Z=\frac{counts-{\mu}_{NL}}{\sigma_{NL}} $$

in which μ_NL_ and σ_NL_ are the mean and standard deviation of PET counts within the normal volume for each study.

The *Z*-score provided a statistical way to standardise relative intensity of FDG uptake throughout the lungs. All lung voxels exceeding a defined *Z*-score threshold were defined as part of FDG-avid lesions. We then exported images of segmented lesion volumes to view alongside the original images for visual quality assessment.

**Fig. 3:**
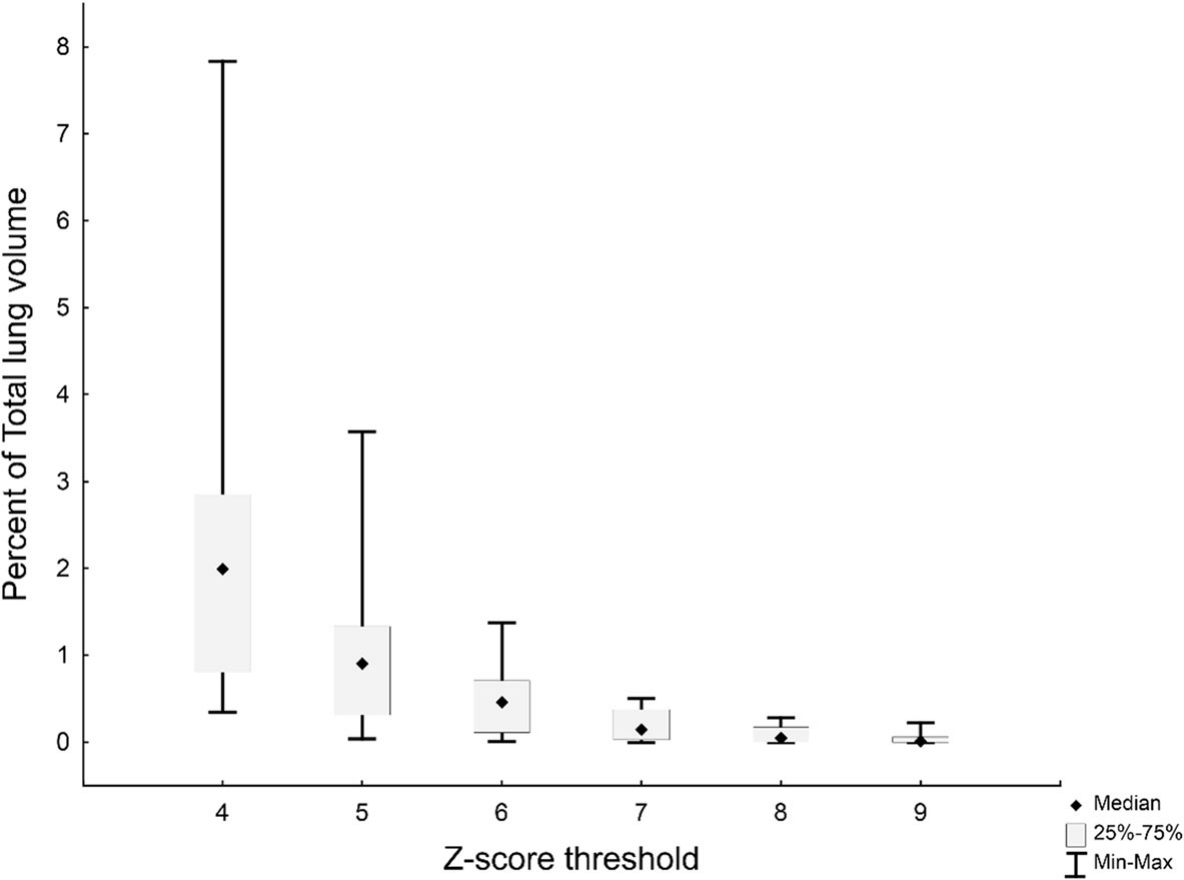
The range, median and 25th and 75th percentiles for percentage of total lung volume classified as FDG avid at different *Z*-score thresholds [4–9], in the quantification of PET-CT scans from 15 controls with visually lesion-free lungs

**Fig. 4:**
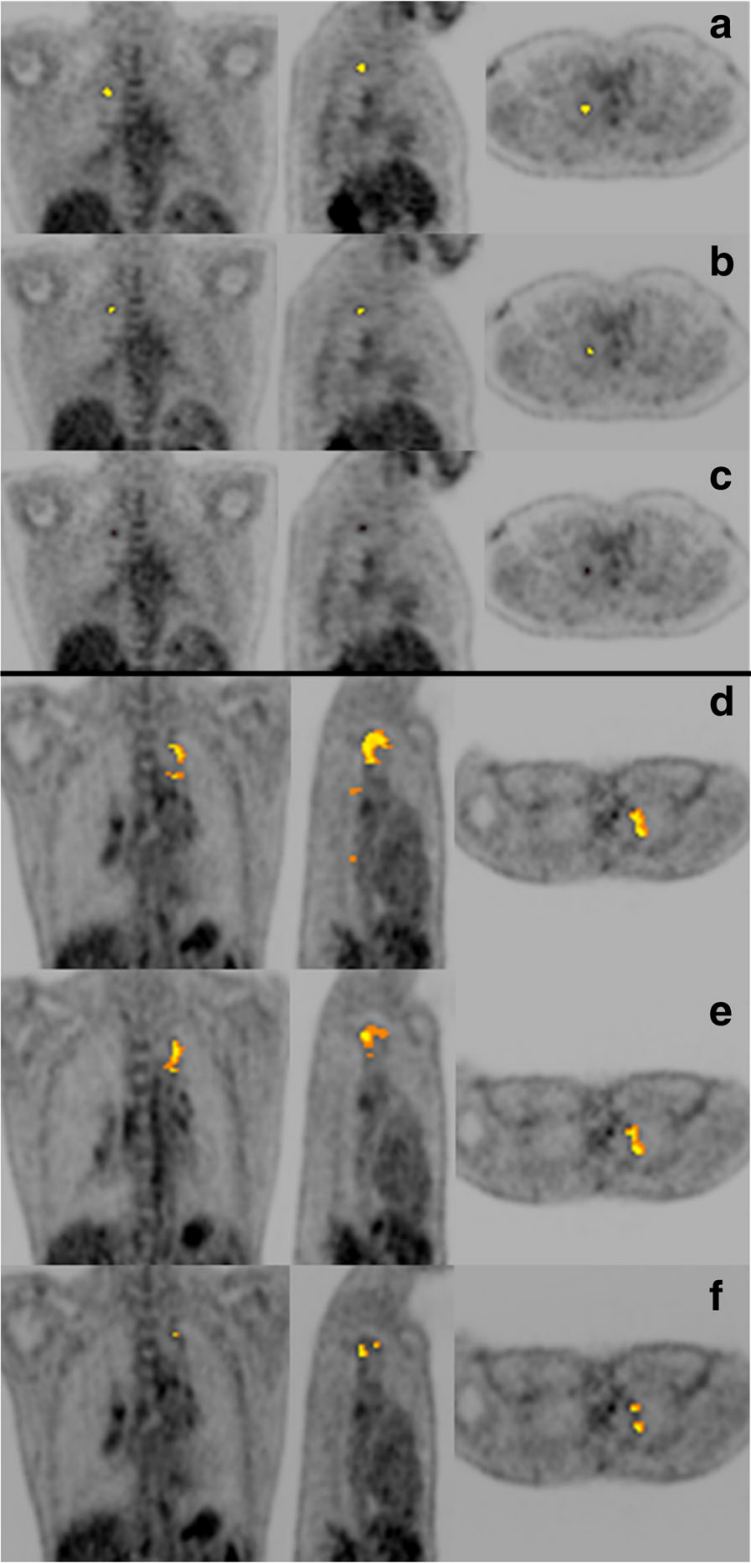
Coronal (left), sagittal (middle) and transverse views of PET scans of two patients after treatment for pulmonary tuberculosis. The first patient (**a**–**c**) has a residual nodule in the right upper lobe showing minimally increased FDG avidity. The second patient (**d**–**f**) has a complex lesion in the left upper lobe with mild FDG avidity. The auto-delineated metabolic lesion volume is overlaid using ascending *Z*-score thresholds, respectively at *Z* = 7 (**a**, **d**), *Z* = 8 (**b**, **e**) and *Z* = 9 (**c**, **f**)

To determine the optimal *Z*-score threshold, we processed a set of 15 lesion-free lung scans with reiterations of increasing *Z*-score thresholds, to minimise false positive findings. As expected, at a low *Z*-score threshold, the volumes segmented as abnormal in these healthy lungs were high, but decreased to a value close to zero for *Z* ≥  8 (Fig. 3). To minimise false negative findings, we also tested increasing *Z*-score thresholds on scans that showed residual lesions with minimal or mild intensity and complex morphology, obtained from five PTB patients after treatment. Intensity was graded using an adaptation of the Deauville classification [18, 42]. A threshold of *Z* = 8 delineated all lesions with minimal FDG avidity, while *Z* = 9 delineated all lesions with mild FDG avidity, but failed to detect some lesions with minimal FDG avidity (Fig. 4). We thus chose a cut-off of *Z* = 8 based on its low false positive and false negative rates.

For comparison, two readers manually delineated FDG-avid lung lesions of diagnosis (Dx) and M6 scans, based on visual assessment. We also applied different thresholds for automated segmentation within the lung mask, namely SUV > 1 and T40%. SUV > 1 is a fixed threshold, used in previous TB studies [41] to delineate lung lesions on PET, while T40% refers to 40% of the maximum intensity in a VOI, previously used in cancer studies [6].

#### CT density quantification

Concurrently with the PET segmentation and using the same lung mask, the MATLAB script also segmented the CT images into five categories based on the density of each voxel compared to set values obtained from literature [21, 41]: (1) low density (< − 950 HU), attributed to cavitation or extremely hyper-inflated lung tissue (*V*_low_); (2) normal density, between − 950 HU and − 500 HU; (3) soft lesion volume (*V*_soft_), from − 500 HU to − 300 HU, usually tree-in-bud lesions or nodules, but may also include regular, medium to large vasculature; and (4) medium lesion volume (*V*_medium_) from − 300 HU to − 100 HU. This category should include very little normal lung tissue and usually consists of nodular infiltrates, but may also include hard lesions in early progression or partial resolution; (5) hard lesion volume (*V*_hard_), above − 100 HU, are usually due to consolidation, cavity walls, bronchial thickening or calcified fibrosis.

We performed visual checks of the accuracy of CT lesion delineation, based on these fixed density thresholds. For lesions with increased density (*V*_soft_, *V*_medium_, *V*_hard_), the segmented areas corresponded well to lesion morphology. However, using − 950 HU as the upper limit for low-density lesions was not specific enough for cavitation and the segmented areas in some cases included bullae, bronchiectasis, and severe emphysema. This necessitated an additional step to measure the volumes of individual cavities for each scan. This was done using the MRICro’s 3D region-growing tool (boundary-based segmentation).

#### PET lesion intensity quantification

After segmentation, the program quantified the following PET parameters: (1) MLV, (2) the mean *Z*-score in the MLV (*Z*_mean_), (3) TGA (MLV × SUV_mean_), (4) total glycolytic activity index (TGAI): the product of the MLV and mean lesion-to-background intensity (*TGAI* = *MLV* × *mean lesion counts*/*mean counts in normal lung*). In addition, the program also measured the total lung volume [TLV (ml)] and the volumes of each abnormal density category on CT, i.e., *V*_low_, *V*_soft_, *V*_medium_, and *V*_hard_, as well as a combined PET-CT metric: MLV_abN_ [the intersection of MLV and area with increased density on CT (≥ 500 HU)].

Re-slicing of the CT to the corresponding PET allowed the program to compare CT density and PET intensity, directly per voxel.

#### Manual segmentation

The two manual readers are clinicians, with experience in the diagnosis and treatment of pulmonary tuberculosis. They are also clinical trial investigators and trained in PET-CT evaluation for a phase 3 clinical trial that aims to use PET-CT parameters to guide TB treatment duration [28]. Manual segmentation of FDG-avid PET lesions in PTB scans took roughly 30–90 min per scan.

#### Statistical analysis

Volumetric comparisons were performed using the dice similarity coefficient (DSC), a validated approach to measure spatial overlap [51, 52]. In addition, the true positive volume fraction (TPFV) was calculated to indicate segmentation sensitivity and one minus the false positive volume fraction (1-FPVF) to indicate specificity. Statistical analysis was performed using Statistica Version 13©. For numerical variables we report median, range and 25th and 75th percentiles to accommodate the modest sample size included in this pilot project. Agreement measures are reported using a Bland-Altman plot showing only bias and values and not confidence intervals and standard deviations. We report the correlation coefficient and *p* values for correlations between numerical variables.

## Results

### Application of technique

We successfully implemented the quantification methodology in all controls and cases. Creating the masks required knowledge of lung anatomy, while the other steps required basic computer literacy. The user input required for quantification could be divided into (1) file management (including the selection, indexing and formatting of image files) and (2) creating VOIs for lung masks, background references and areas affected by motion misregistration. The time required to quantify scans ranged approximately from 10 min for a lesion-free lung scan at one time-point, to 45 min for a three time point series of extensively diseased lungs. Where applicable, cavity volumes were easily measured with MRICro’s 3D region-growing tool on CT, taking less than 1 min per scan.

The auto-segmented MLV for each scan corresponded well with visual assessment of the PET scans. No false positive segmentation was noted on control scans. In the PTB lung scans, all auto-segmented MLVs corresponded to areas that appeared FDG avid. No visually FDG-avid lesions were missed by auto-segmentation.

FDG-avid lesions were also segmented using T40%. However, when max lesion SUV decreased during treatment, the MLVs became progressively more inclusive and did not correspond well to visual assessment. As such, we abandoned T40% as a method to monitor treatment response in a whole lung VOI.

Table 2 shows a summary of DSC values (Table 2), true positive volume fraction (TPVF; Table 2), 1-false positive volume fraction (1-FPVF; Table 2), between readers and automated thresholds at Dx, M6 and combined. Individual segments corresponded better at Dx than M6, independent of which segmentation method used. DSC between manual and *Z*-score segmentations (Z-A to reader A and Z-A to reader B) was slightly lower than between the manual readers (reader B to reader A), but higher than DSC between manual and SUV > 1 segmentations. The same trends were noted in regard to 1-FPVF(%) values. In contrast, SUV > 1 segments performed better in regard to TPVF comparison than either *Z*-score or manual segmentation. The same behaviour was also observed when comparing to either unions or the intersections. The DSC between MLV segmented using the *Z*-score and the MLV intersection between the readers was 0.73 (range 0.46–0.94), while the DSC between the readers’ MLV union was 0.72 (range 0.3–0.94). The median DSC between the MLV segmented using SUV > 1 and the readers’ segments intersection was 0.61 (range 0.11–0.88) and 0.7 (range 0.18–0.92) when compared to the union of the manual segments.

**Table 2:** Summary of comparisons between independent readers, reader A and reader B, and automated thresholding segmentations using the *Z*-score (Z-A and Z-B) and SUV > 1 (SUV). Values for dice similarity coefficient (DSC), true positive volume fraction (TPVF) and 1 minus false positive volume fraction (1-FPVF) are shown

	Dx and M6		Dx		M6	
	Median	Range	Median	Range	Median	Range
DSC						
Z-A to reader A	0.75	0.57–0.91	0.85	0.57–0.91	0.68	0.61–0.84
Z-A to reader B	0.66	0.31–0.94	0.77	0.56–0.94	0.55	0.31–0.79
Z-A to intersection	0.73	0.46–0.89	0.81	0.49–0.89	0.64	0.46–0.78
Z-A to union	0.72	0.30–0.94	0.81	0.63–0.94	0.55	0.30–0.85
SUV to reader A	0.68	0.21–0.90	0.74	0.57–0.90	0.47	0.21–0.77
SUV to reader B	0.63	0.19–0.91	0.75	0.57–0.91	0.45	0.19–0.71
SUV to intersection	0.61	0.11–0.88	0.74	0.50–0.88	0.45	0.11–0.69
SUV to union	0.7	0.18–0.92	0.77	0.64–0.92	0.37	0.18–0.79
Z-A to SUV	0.88	0.15–0.99	0.88	0.75–0.97	0.59	0.15–0.99
Z-B to Z-A	0.83	0.59–0.99	0.89	0.79–0.99	0.63	0.59–0.95
Reader B to A	0.78	0.42–0.92	0.83	0.78–0.92	0.64	0.42–0.85
TPVF (%)						
Z-A to reader A	91.4	55.48–100.00	98.9	89.21–100.00	78.5	55.48–93.65
Z-A to reader B	80.5	18.22–99.91	96.3	70.18–99.91	62.0	18.22–94.05
Z-A to intersection	98.0	59.56–100.00	99.9	94.35–100.00	87.5	59.56–98.56
Z-A to union	77.8	17.86–99.92	96.0	70.82–99.92	77.8	17.86–90.34
SUV to reader A	99.4	34.64–100.00	99.8	99.34–100.00	96.6	34.64–100.00
SUV to reader B	92.0	31.92–99.63	96.7	72.27–99.63	84.8	31.92–96.95
SUV to intersection	100.0	53.19–100.00	100.0	99.83–100.00	99.8	53.19–100.00
SUV to union	95.4	24.64–99.91	96.4	73.04–99.91	95.3	24.64–97.28
Z-A to SUV	91.4	17.09–100.00	87.9	59.49–100.00	97.5	17.09–100.00
Z-B to Z-A	92.7	56.35–100.00	86.9	70.80–100.00	100.0	56.35–100.00
Reader B to A	81.4	50.25–98.10	88.5	79.06–98.10	71.1	50.25–93.15
1-FPVF(%)						
Z-A to reader A	68.8	40.61–88.04	74.5	40.61–84.70	61.3	55.79–88.04
Z-A to reader B	68.3	31.79–100.00	74.4	39.29–91.24	52.9	31.79–100.00
Z-A to intersection	62.7	30.95–88.04	70.8	32.51–79.93	47.9	30.95–88.04
Z-A to union	75.7	30.54–100.00	88.3	46.25–92.29	62.2	30.54–100.00
SUV to reader A	57.7	11.82–81.57	59.2	39.99–81.57	54.4	11.82–73.02
SUV to reader B	54.6	10.97–85.55	74.6	40.51–85.55	42.8	10.97–70.14
SUV to intersection	48.6	6.10–78.25	58.2	32.94–78.25	30.2	6.10–69.07
SUV to union	66.0	9.91–88.88	75.7	46.85–88.88	55.2	9.91–74.10
Z-A to SUV	100.0	8.36–100.00	100.0	77.01–100.00	100.0	8.36–100.00
Z-B to Z-A	87.3	44.02–100.00	90.3	86.36–100.00	61.5	44.02–89.62
Reader B to A	77.3	26.93–95.40	77.7	65.93–95.40	59.1	26.93–95.32

**Fig. 5:**
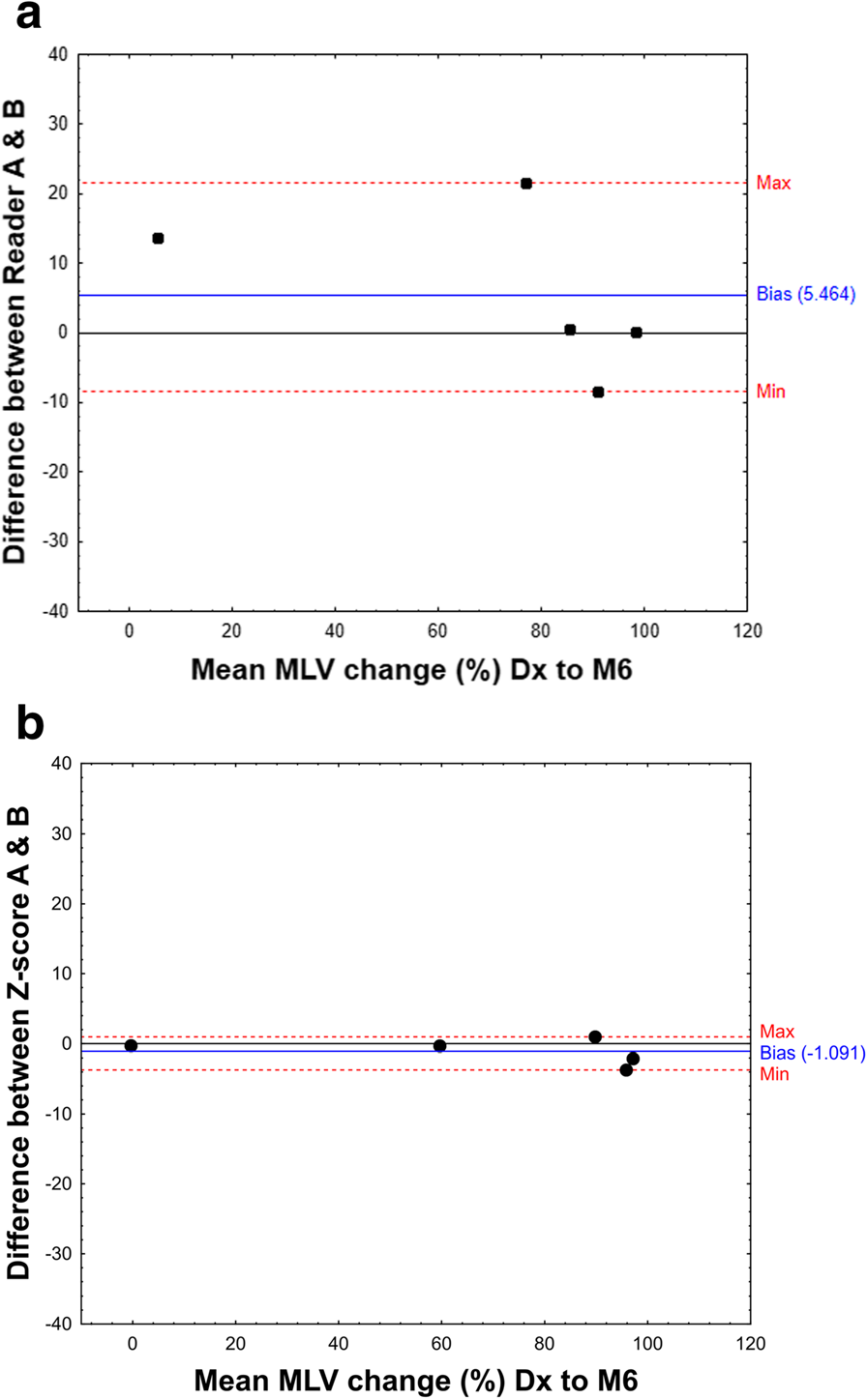
Bland-Altman plots to show inter-user agreement between the mean percentage change in metabolic lesion volume from diagnosis to month 6 of treatment. **a** Manual segmentation of reader A and reader B. **b** *Z*-score segmentation created using normal lung and lung masks defined by user A and user B

Inter-user *Z*-score segmentation (created by different spheres and lung mask) and inter-reader manual segmentation showed similar variability at single time-points, with a median DSC of 0.83 and 0.78 respectively (Table 2). However, there was a much better agreement in percentage change from diagnosis to M6 for inter-user *Z*-score segmentation than for manual inter-reader segmentation. The difference between the percentage change from Dx to M6 in MLV lesions volume ranged from − 3.69 to 0.96 for inter-user *Z*-scores and − 8.44 to 21.51 for inter-reader MLV change (Fig. 5).

**Fig. 6:**
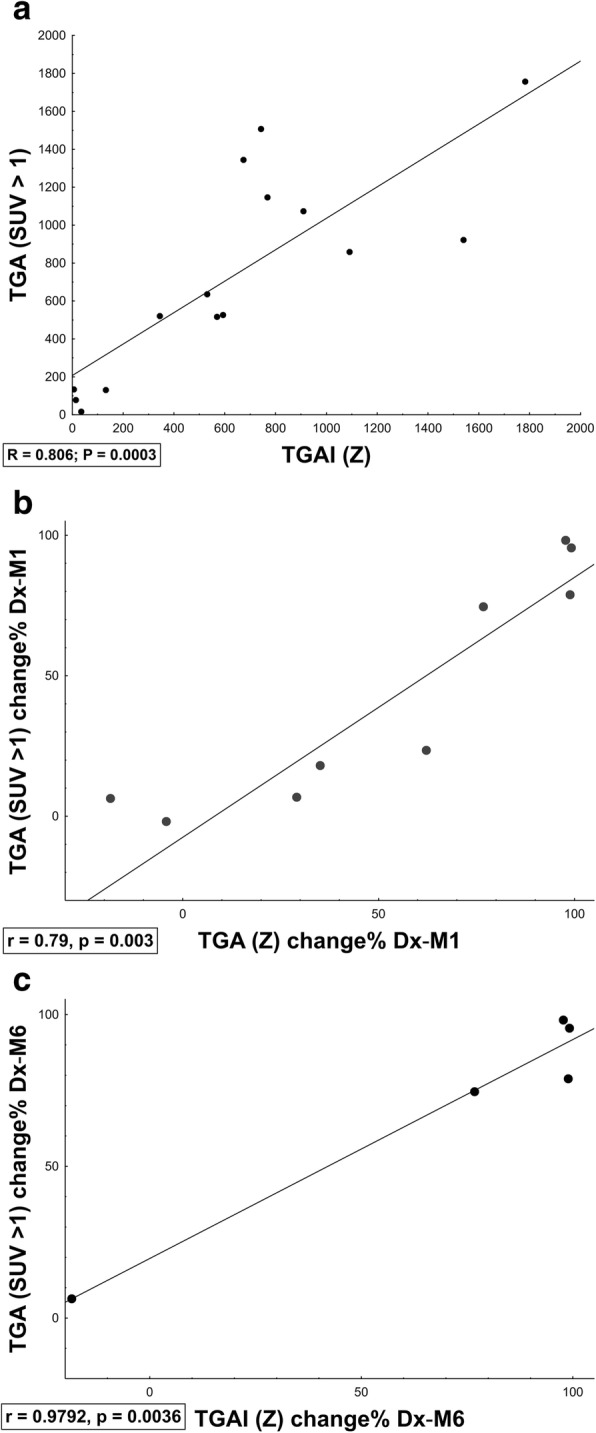
Scatter plots showing correlation between the TGA (based on SUV and MLV determined by SUV > 1) and TGAI (based on lesion-to-background ratio and MLV created by *Z*-score). **a** Total values at Dx, M1 and M6. **b** Percentage change from Dx to M1. **c** Percentage change from Dx to M6

*Z*-score segmentation correlated well with SUV > 1 (median DSC = 0.88). In addition, there was a high correlation between TGA (product of SUV_mean_ and MLV in SUV > 1) and TGAI (product of mean lesion-to-background intensity and MLV in *Z*-score > 8) across time-points (Dx, M1, M6, Fig. 6a), which improved when assessing change in inflammatory burden over time (Fig. 6b, c).

### Response to therapy

**Fig. 7:**
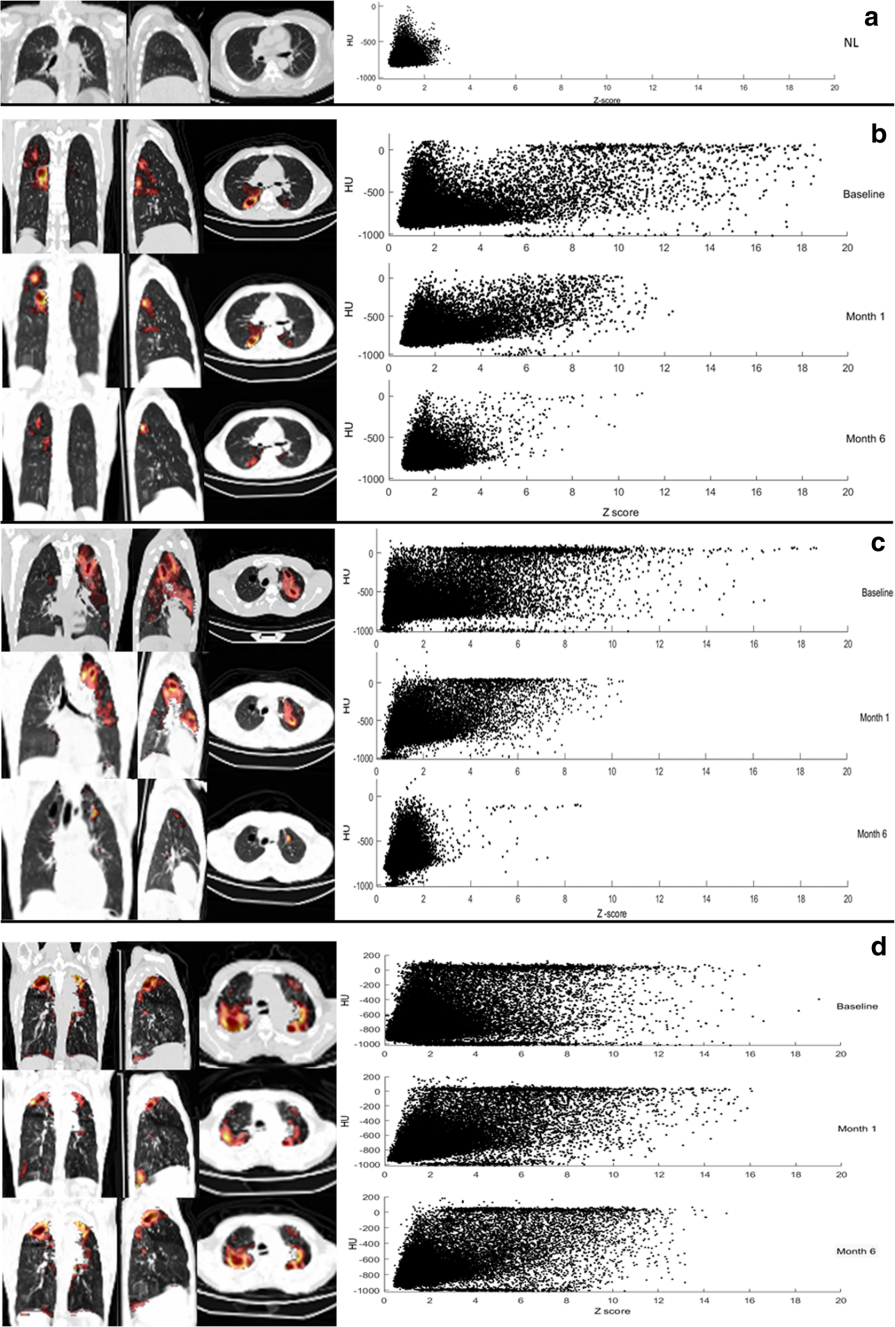
This shows coronal (left), sagittal (middle) and transverse (right) views of different CT images with the auto-delineated metabolic lesion volume as an overlay and a scatterplot representing the CT density (*Y* axis, HU) and the PET uptake intensity (*X* axis; *Z*-score) of the voxels held within the lung mask **a** Lesion-free lungs from control participant. **b**–**d** Dx, M1 and M6 PET-CT scans for PTB patients. **b** Cured patient with an improved scan response and moderate uptake still present at M6. **c** Cured patient, with a resolved scan response. Only minimal intensity residual lesions and thin-walled cavities still present at M6. **d** Failed treatment case, with a mixed scan response pattern and multiple lesions with very high uptake present at M6

Representative quantification patterns of scans from a control case and PTB patients before, during, and after treatment, with an overlay of the segmented MLV and a scatterplot representing the voxels from each scan, are shown in Fig. 7. The scatter plot of a control scan is shown in Fig. 7a. The cured patients’ values (Fig. 7b, c) change toward normal at follow-up, while for the patient who failed treatment the pattern remains clearly abnormal on the density and intensity axes (Fig. 7d).

**Fig. 8:**
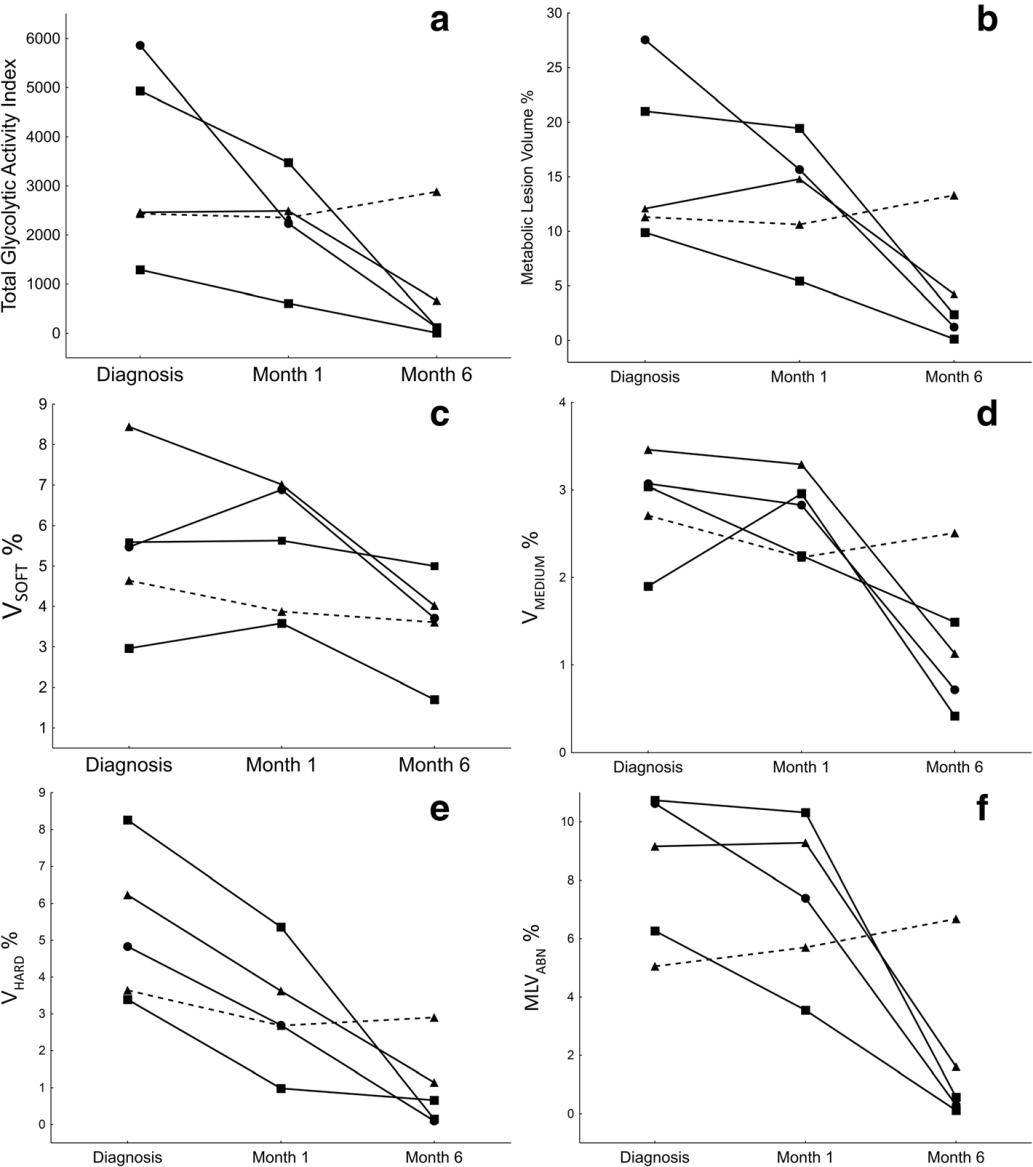
These graphs represent the case-profiles of five different PTB patients and the changes of various auto-segmented PET-CT parameters at Dx, M1 and M6 (*X* axis). A straight line represents a cured outcome and the broken line indicates treatment failure. **a** Total glycolytic activity index. **b** Metabolic lesion volume as a percentage of total lung volume. **c** *V*_soft_ (− 500 HU:− 300 HU) CT lesion volume as a percentage of total lung volume. **d** *V*_medium_ (− 300 HU:− 100 HU) CT lesion volume as a percentage of total lung volume. **e** *V*_hard_ (> − 100 HU) CT lesion volume as a percentage of total lung volume. **f** Area of lung that shows abnormal density (> − 500 HU) and relative high uptake intensity as a percentage of total lung volume

Figure 8 demonstrates the dynamics of different scan parameters during treatment of the five PTB cases, in relation to clinical outcome. The segmented TGAI (Fig. 8a) of three out of four cured cases already showed partial reduction (30–62%) at M1 and continued to decrease markedly toward the end of treatment. The fourth cured patient showed a slight increase by M1, but significant reduction by M6. After 6 months of treatment, all the cured patients showed marked reduction (80–99%), but only one patient showed nearly complete metabolic resolution. The patient that failed treatment remained unchanged over the first month and deteriorated by M6. The MLV for this patient (Fig. 8b) showed very similar patterns during treatment.

The combined lung volume with increased density (*V*_soft + medium + hard_) followed a similar trend, although the decrease from Dx to M6 was less marked (41–83%) than MLV changes and the failed treatment patient showed only slightly decreased values from Dx to M6. In the breakdown of lesions of different density (Fig. 8c–e), all cured patients showed a marked volume decrease in hard lesions, already noted by M1 (35–71% reduction) and continued toward M6 (81–98% reduction). The volumes of medium and soft lesions were more variable, and a relatively large residual lesion volume was present at M6 for most cases.

After 1 month of treatment, in cured patients, MLV_abn_ (Fig. 8f) decreased modestly in two and remained stable in the other two. The failed treatment case showed a 13% increase. At M6, however, all cured cases’ MLV_abn_ had decreased markedly (82–98%), while the failed treatment case showed a further increase.

## Discussion

In this paper, we present a technique to reproducibly quantify pulmonary tuberculosis lesions on PET and CT. We used patient-specific reference volumes to reduce intra- and interscan variability on PET and auto-delineation to reduce inter-reader and inter-user variability when assessing multiple widely distributed lesions with complex morphology. The technique quantifies lesions throughout the lungs in order to measure the central trend of disease progression or resolution, thus compensating for the variable response of individual lesions during PTB treatment.

The technique introduced some novel concepts in PET-CT analysis including the use of a patient-specific reference volume to standardise lung uptake (lesion-to-background), whole lung automated segmentation of CT lesions and bivariate quantification of PET and CT images. It also reapplies concepts previously used in other settings, such as co-registration of corresponding scans at different time-points, whole organ (semi-)automated segmentation of PET uptake, and joint segmentation of PET and CT [23].

Optimal adaptive thresholds for auto-segmentation of PET scans were determined using a set of control lung scans and scans containing lesions with minimal to mildly increased uptake. Density thresholds for CT scans were determined using values previously reported in literature [41].

We compared the *Z*-score delineation to manual delineation by two independent readers and a fixed threshold previously used in a TB trial (SUV > 1) [41]. There was better correlation between the *Z*-score and manual segmentation when compared to the fixed threshold. We also found decreased inter-user variability when measuring response to treatment when compared to manual segmentation. However, there was still some inter-user variability at single time-points, which would not apply when using a fixed threshold (SUV > 1). Both automated thresholding techniques saved time and appeared highly sensitive. We also applied a gradient-based threshold (T40%); however, it did not appear to be appropriate to measure response to treatment in whole lung VOIs. After treatment, agreement between all thresholding techniques and readers were lower than previous reports only focussing on diagnosis [6]. However, this was expected in highly complex lesions at the very edge of FDG avidity, found after treatment.

There was good correlation between TGA (based on mean SUV and volume) and TGAI (normalised to background lung activity), especially when measuring changes over time.

We tested whether the technique was accurate enough to measure changes over time, by applying it to independent pilot sets of control cases and PTB cases during treatment. The quantified variables detected marked changes within 1 month of treatment. The metrics corresponded well with visual scan interpretation, clinical outcomes and the qualitative classification allocated during analysis for the parent study. It also provided a range of additional information.

Some challenges and potential drawbacks were encountered during this pilot application. While using the 3D region-growing tool in MRIcro was user friendly and easily reproducible, some manual input was required when drawing the lung mask, to include dense lesions extending into the chest wall, and areas of motion misregistration. In the future, computer-assisted drawing tools or fully automated lung atlas segmentation and correction of misregistration should decrease inter-user variability and time requirements of any automated technique.

Co-registering baseline and follow-up scans allowed the user to generate a single-lung mask and reference volume to use across all time points. This reduced interscan variability and saved time. It did not, however, allow for the measurement of changes in total lung volume over time, which may occur after lesion resolution with associated fibrosis. Re-slicing allowed direct voxel comparison of PET and CT components using a single VOI. A disadvantage is that there was a smoothing effect on the CT images. Using a density threshold was not specific enough to delineate cavity volume, and an additional manual step was required to perform this function.

Using the mean and standard deviation of reference volumes to standardise lesion-to-background activity should decrease interscan and inter-patient variability compared to techniques that normalise the uptake to reference volumes from other organs or a theoretical whole body concentration. Thus, the *Z*-score takes into account the variability of FDG uptake in normal lung tissue and does not depend on dose or weight. The advantage of the latter is that changes in weight induce changes in SUV, while not necessarily inducing a change in FDG uptake.

Semi-automatic delineation of the whole lung allowed for segmentation of multiple lesions with widespread distribution and variable intensity, size and morphology. This shows promise to reduce inter-reader variability, especially in a self-controlled study measuring changes after interventions.

## Conclusions

This technique to auto-segment and quantify multi-focal and complex lung lesions on PET and CT images shows great promise in a pilot set of subjects and required limited operator input. This study was only a small pilot study as proof-of-concept. Validation in a larger cohort, and comparison of scan metrics to clinical outcomes and biomarkers is required to determine indicators of prognosis and cure. Ultimately this methodology, or features thereof, could be incorporated into standard clinical analysis pipelines and research protocols.

## Abbreviations

CT: Computed tomography
Dx: Diagnosis
HU: Hounsfield units
M1: Month 1
M6: Month 6
MLV: Metabolic lesion volume with high density (> − 500 HU)
MLV_abn_: Metabolic lesion volume with high density (> − 500 HU) and high intensity (> Z-score 8)
NL: Normal lung parenchyma
PET: Positron emission tomography
PTB: Pulmonary tuberculosis
TGA: Total glycolytic activity (SUV_mean_ × lesion volume)
TGAI: Total glycolytic activity index (mean lesion-to-background × lesion volume)
TLV: Total lung volume
*V*_hard_: Hard lesion volume (> − 100 HU)
*V*_low_: Hypodense lesion volume (> − 950 HU)
*V*_medium_: Medium lesion volume (− 300 HU to − 100 HU)
VOI: Volume of interest
*V*_soft_: Soft lesion volume (− 500 HU to − 300 HU)
*Z*: Z-score/standardised score
*Z*_mean_: Mean standardised intensity
